# The Role of Matrix Metalloproteinases (MMP-8, MMP-9, MMP-13) in Periodontal and Peri-Implant Pathological Processes

**DOI:** 10.3390/ijms23031806

**Published:** 2022-02-04

**Authors:** Ionut Luchian, Ancuta Goriuc, Darius Sandu, Mihai Covasa

**Affiliations:** 1 Department of Periodontology, Faculty of Dental Medicine, “Grigore T. Popa” University of Medicine and Pharmacy, 16 Universității Street, 700115 Iasi, Romania; ionut.luchian@umfiasi.ro (I.L.); darius-valentin.lr.sandu@students.umfiasi.ro (D.S.); 2 Department of Biochemistry, Faculty of Dental Medicine, “Grigore T. Popa” University of Medicine and Pharmacy, 16 Universității Street, 700115 Iasi, Romania; 3 College of Medicine and Biological Sciences, University “Stefan cel Mare” Suceava, 13 Universității Street, 720229 Suceava, Romania; mcovasa@usm.ro; 4 Department of Basic Medical Sciences, College of Osteopathic Medicine, Western University of Health Sciences, 309E Second Street, Pomona, CA 91766, USA

**Keywords:** periodontitis, periodontal disease, MMP-8, MMP-9, saliva, crevicular fluid

## Abstract

Severe periodontitis, a destructive inflammatory disease of the supporting tissues of the teeth, ranks sixth in terms of global spread, affecting about 11% of the population. Metalloproteinases (MMPs) are extracellular matrix (ECM) macromolecules that are important in cellular development and morphogenesis, and they are capable of activating growth factors in their proximity, cell surface receptors, and adhesion molecules. MMPs are part of a major family of zinc-dependent endopeptidases, and their activity is modulated and regulated by certain inhibitors known as tissue metalloproteinase inhibitors (TIMPs). Because type I collagen is the major component of the periodontal extracellular matrix, special attention has been paid to the role of collagenases, especially MMP-8 and MMP-13 and gelatinases, MMP-2 and MMP-9, in periodontal diseases. In fact, MMP-8 (or collagenase 2) is currently one of the most promising biomarkers for periodontitis in oral fluids. Among them, salivary MMP-9 has been shown to be a more sensitive marker for periodontal inflammation during orthodontic treatment, which opens new perspectives in reducing periodontal hazards during such treatments. Both MMP-8 and MMP-9 are extremely valuable diagnostic tools in treating periodontitis, and future studies and healthcare policies should focus on implementing more accessible methods of chairside testing in order to reduce the prevalence of this disease.

## 1. Introduction

Severe periodontitis, a widely spread severe inflammatory disease leading to the destruction of the supporting tissues of the teeth, affects approximately 11% of the global population [1]. It is estimated that this condition causes losses of billions of dollars globally each year, both through high direct costs of treatment and indirectly by affecting the condition of patients [2]. However, periodontitis treatment is relatively simple and cost-effective if diagnosed in the early stages. Quite challenging, periodontitis is usually asymptomatic, meaning that a person with the disease is unaware of it, and its traditional diagnosis requires specialist doctors and equipment that is often not available, especially in poor environments [3,4].

The development of microorganisms at the dental level (the main cause of periodontal damage) involves a wide range of macromolecules, including many proteins produced by different cells. These proteins include matrix metalloproteinases (MMPs) involved in the degradation of the extracellular matrix and whose functions are modulated by tissue metalloproteinase inhibitors called TIMPs. MMPs (or matrixins) are an important family of calcium-dependent zinc-containing endopeptidases; they are able to degrade not only extracellular matrix proteins but also non-matrix proteins, including cytokines [5]. Degradation of extracellular matrix proteins (ECMs) by proteinases is a key feature of periodontal disease and can be derived from both microorganisms in dental plaque and from cellular sources [6]. Previous studies have shown that proteases derived from both microbial and cellular sources can contribute to the activation cascade of MMPs, leading to periodontal destruction [7]. Moreover, microbial proteases stimulate proteolytic activators of latent human pro-MMPs, which can increase their secretion by gingival resident cells, causing the degradation of ECM collagen.

MMPs are involved in multiple pathological processes in the body, including oncological pathology [8]. Some metalloproteinases are involved in metastases, others are associated with the level of tumor aggression, and some of them are either used as markers for the diagnosis of certainty or for prognosis determination. For example, MMP-2 is involved in hard dental tissues, favoring a dental tissue much more sensitive to karyogenic acid attacks, by degrading the enamel proteins such as amelogenin [9]. Increased activity of MMP-2 and MMP-9 has been observed in apical lesions following pulpal necrosis, which may demonstrate their involvement in the development of apical periodontitis. Given that numerous studies have shown an increased expression of MMP-9 and MMP-8 activity in patients with periodontal disease, they have been proposed as valid indicators of the disease [10]. For example, in patients subject to orthodontic treatment, high levels of MMPs, especially MMP-2 and MMP-9, have been found at the sites of tension and compression of the teeth undergoing orthodontic treatment [11].

### Search Methodology

We used PubMed, Scopus, Embase, Web of Science, and Google Scholar databases for keywords: matrix metalloproteinases, periodontal disease, periodontitis, and peri-implantitis and retrieved only human clinical research papers and reviews that were written in English. Observational studies were excluded from analyses as well as those that did not meet these criteria after reading the abstract.

## 2. Matrix Metalloproteinases and Their Physiopathological Involvement

The MMPs were first described as early as 1949 with the discovery of depolymerizing enzymes involved in connective tissue growth [12]. However, the research into what is currently known as the family of matrix metalloproteinases began in 1962 when Woesnner, followed by Gross and Lapiere, discovered and characterized an enzyme with collagenolytic activity in the amphibian tissues [13]. It was only in 1980 that Harris et al. proposed using the name MMPs for this group of collagenases/gelatinases. The past 60 years have seen remarkable progress in studying the biological functions of MMPs and their involvement in numerous biological processes such as tissue repair and remodeling, cellular differentiation, embryogenesis, morphogenesis, cell proliferation, apoptosis, would healing, and reproduction, to name a few [14]. Not surprisingly, the deregulation of MMPs activities leads to several pathological processes and diseases such as periodontal diseases, arthritis, cancer, neurodegenerative disorders, cirrhosis, and cardiovascular abnormalities.

MMPs (or matrix metalloproteinases) are major enzymes involved in extracellular matrix remodeling; they can also act intracellularly, are capable of activating growth factors in their proximity, cell surface receptors, and adhesion molecules [15], and are classified based on the specificity of the substrate. Despite the fact that they largely represent a sequence of similar structures, there are still differences in the substrate specificity [16] represented by degraded matrix proteins (Table 1). Currently, there are 28 members in this family, with 25 of them present in humans [8,9]. They are described and classified into five sub-families based on their function and structure: collagenases, gelatinases, stromelizines, membrane matrix metalloproteinases (MT-MMPs), and other MMPs [14]. Some of the domains in the structure of MMPs are indispensable for the intracellular transport of secreted enzymes to the cell membrane, which are finally eliminated after the protease is secreted. MMPs also have a domain with latent enzyme capacity. 

The family of metalloproteinases with a particularly important role in numerous physiological and pathological processes at the tissue level is, in fact, represented by a group of enzymes that participate in cleaving the components of the extracellular matrix. Given their role in tissues, they are represented by collagenases, including MMP-1, MMP-8, MMP-13, and MMP-18, which can degrade interstitial collagen I, II, III, resulting in degraded collagen or gelatin. MMP-1, which is synthesized by macrophages, fibroblasts, and dentritic cells, is involved in promoting cell survival. On the other hand, MMP-8, secreted by neutrophils, has antitumor action and anti-invasive properties due to its role in regulating hormone receptors [26].

Gelatinase A (MMP-2) and gelatinase B (MMP-9) are two proteins in this family that are responsible, among other things, for the degradation of type IV collagen in the basement membrane [16]. Gelatinase A (MMP-2), with a molecular weight of 72 kDa, is involved in the degradation of type I, II, and III collagen and, under normal conditions, is expressed by stromal cells in most tissues (hematopoietic, endothelial, dendritic cells, fibroblasts, mast cells, and macrophages). Gelatinase B (MMP-9) has a molecular weight of 92 kDa, is found in very small amounts in normal tissues, and is secreted by dendritic, hematopoietic, macrophage, neutrophil, fibroblast, and lymphocyte cells [27].

MMP-3, MMP-10, and MMP-11 are also called stromelizines and are involved in the digestion of certain molecules in the extracellular matrix and the basement membrane. MMP-3 indirectly modulates cell migration and is secreted by fibroblasts, lymphocytes, endothelial, and dendritic cells. MMP-11 is located mainly in the adipose tissue in the proximity of a tumor, being correlated with adipogenesis processes. MMP-11 is also a negative modulator of pre-adipocyte differentiation and reverses the differentiation of mature adipocytes, which leads to the peritumoral accumulation of fibroblast-like cells, thus favoring tumor progression [28]. Matrilizines MMP-7 and MMP-26 are the simplest proteins of this family from a structural point of view because they do not present a domain. They act on cell surface molecules and are expressed mainly by tumor cells of epithelial origin. MMP-7 is secreted by macrophages, endothelial cells, and osteoclasts, thus being involved in inflammatory processes, cell invasion, and angiogenesis [17]. Membrane matrix metalloproteinases are part of the basement membrane and modulate the proteolytic activity of other MMPs. They can be divided into transmembrane proteins that bind through a hydrophobic region, such as MMP-14, MMP-15, MMP-16, MMP-24, and proteins with a glycolphosphatidylinositol group (GPI), such as MMP-17 and MMP-25.

**Figure 1:**
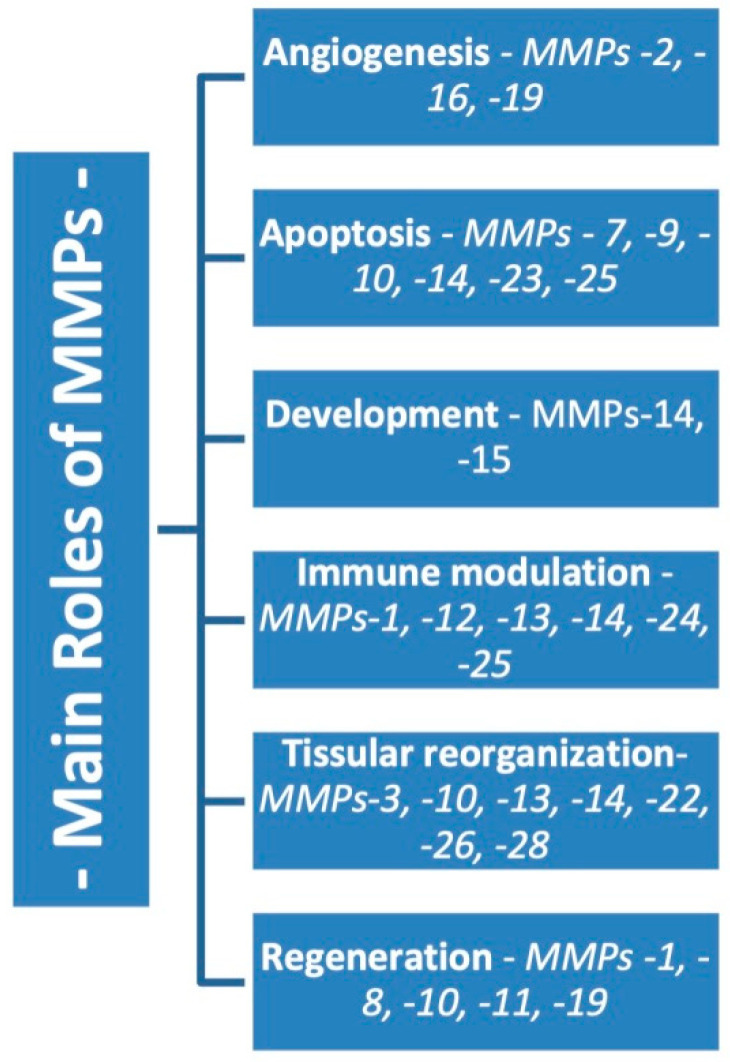
The main functions of matrix metalloproteinases.

The most studied matrix metalloproteinase is MMP-14, which is expressed by fibroblasts, macrophages, endothelial cells, and hematopoietic cells. They are involved in cell growth and in stimulating adipogenesis and angiogenesis. In addition to metalloproteinases, which are grouped according to their structure and role, there are the MMPs, themselves representatives of a family. One such example is MMP-12, a metalloelastase that is synthesized by macrophages with elastin as a substrate and whose migratory capacity is affected [29]. Another metalloproteinase called enamelizine or MMP-20 has, as substrate members of the amelogenin family, extracellular matrix proteins. MMP-28 is expressed in keratinocytes and plays a very important role in hemostasis and wound healing. In addition to all these MMPs with known and well-defined functions, there is also MMP-22, whose functions are not well known, and MMP-23, which is predominantly expressed in reproductive tissue and which does not present the hemopexin domain [27]. (Figure 1).

The activity of metalloproteinases is modulated by tissue inhibitors of metalloproteinases-TIMPs, which comprise four types. An example of such a molecule is TIMP-1, secreted by neutrophils, lymphocytes, and mast cells that can inhibit most MMPs, except for membrane-type metalloproteinase MT1-MMP and MMP-2 [28]. TIMP-2 is secreted by hematopoietic, dendritic, and endothelial cells and inhibits the activity of most MMPs, except MMP-9. TIMP-3 inhibits the activity of MMP-1, -2, -3, -9, and the membrane-type metalloenzyme MT1-MMP [18]. All these are also synthesized by macrophages and fibroblasts.

**Figure 2:**
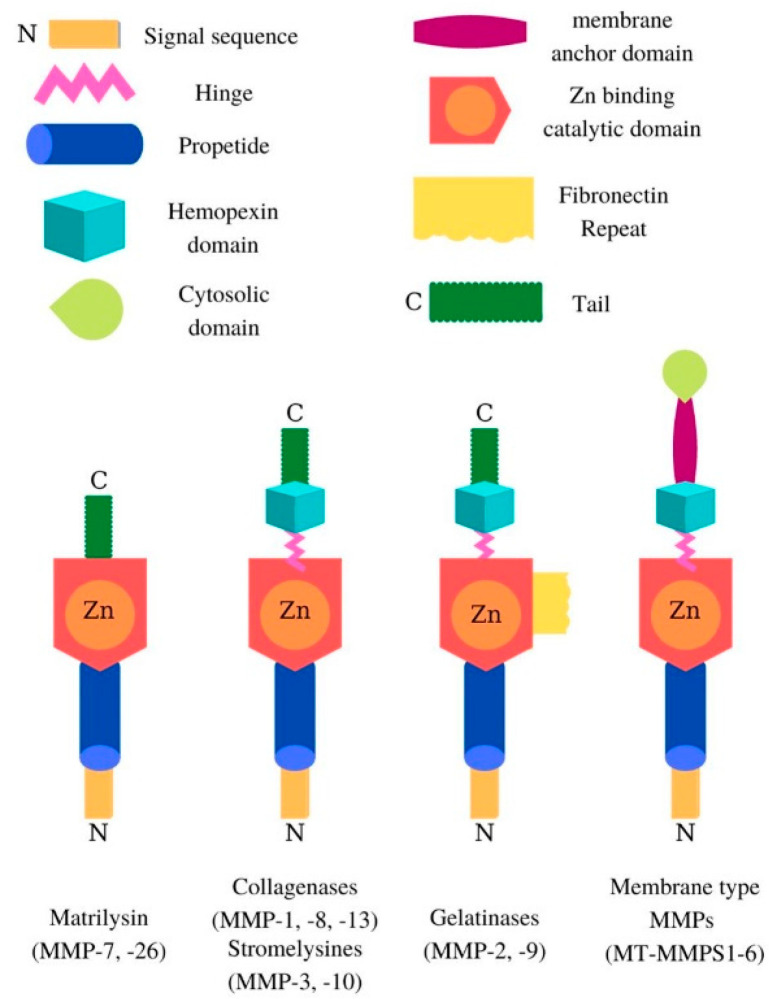
Modular organization of the MMP domain.

As previously mentioned, matrix metalloproteinases play a very important role in various physiological and pathological processes, being responsible for the degradation of extracellular matrix components such as fibers (collagen, elastin, laminin, and fibronectin) and the degradation of proteoglycans and polysaccharides [9,17]. These fibers are mostly collagen-containing glycoproteins, which are considered the main protein in the extracellular matrix. There is a common structure for most members of the metalloproteinase family that comprises a pro-peptide consisting of 80 amino acids with a variable-length N-terminal signaling peptide end, a catalytic domain containing a Zinc ion (170 amino acids), the hemopexin domain involved in collagen degradation (200 amino acids), and a region represented by a variable-length binding peptide, linking the catalytic domain to the hemopexin domain [30] (Figure 2). Despite the many similarities between the members of the MMP family, there are also differences. For example, MMP-7, -23, and -26 do not have the binding peptide or the hemopexin region, while MMP-14, -15, -16, and -24 also have a C-terminus end. MMP-23 exhibits a cysteine arrangement and an Ig-like domain instead of the binding region and the hemopexin domain [31].

In general, the activity of MMPs is closely linked to the existence of a balance between the levels of MMPs and TIMPs, and any alteration of this balance can lead to the progression of the disease/inflammation [32]. Four endogenous inhibitors present in humans, called TIMP-1, TIMP-2, TIMP-3, and TIMP-4, are involved in modulating the activity of MMPs. They have a 40% similar structure, with the difference being represented by the specificity of the substrate. These inhibitors have a role in restoring the components of the extracellular matrix, tissue, and cell remodeling, all of which are mediated by the MMPs themselves [29,32]. TIMPs are inhibitors with high specificity due to the chelating action of Zn2^+^ in the structure of the catalytic domain of MMPs, thus regulating the enzymatic activity of MMP. This complex (MMP-TIMP) is not affected by denaturation through heating or proteolysis [33]. MMPs are involved in various biological processes, including cell proliferation, tissue repair, differentiation, migration, healing, morphogenesis, angiogenesis, and apoptosis (Table 1).

An imbalance in the regulation of MMPs activity can lead to tissue destruction, fibrosis, and degradation of the extracellular matrix, which represent various stages of the disease progression, including periodontal disease [14]. The MMPs degrade the components of the extracellular matrix, the basement membrane, and certain enzymes (SERPINs—serine protease inhibitors), which have a great impact on cytokines, osteoclast activation, tissue regeneration, and loss of connective tissue attachment. The fibroblasts that are part of the desmodontium and also the gingival cells synthesize collagenases such as MMP-1, -8, and -13. On the other hand, MMP-2 and MMP-9 are mainly synthesized by neutrophils and macrophages and are involved in MMPs-mediated destructive periodontal disease. Increased amounts of these MMPs are released from epithelial cells, which can influence apical migration and lateral extension of the junctional epithelium. Finally, these processes lead to the loss of connective tissue attachment [34].

Although many studies have shown that several MMPs contribute to the progression of periodontal disease, such as MMP-2, -7, and -14, the most reported MMPs that are responsible for periodontopathy are collagenases MMP-1, -8, and -13. This group of collagenases has the ability to degrade almost all types of collagenous and non-collagenous proteins in the extracellular matrix. Previous studies have shown that MMP-1 and MMP-8, in addition to being intensively involved in periodontal pathology, are also strongly correlated with cardiovascular pathology and diabetes [35]. Table 1 highlights the substrate, production, and physiological functions and the associations with other collagenase-induced diseases, such as MMP-1, -8, and -13.

## 3. The Relationship between MMPs and Periodontal Disease

*Treponema denticola*, considered a periodontal pathogen, secretes certain proteases that activate pro-MMP-2, released in its inactive form by the periodontal ligament cells. MMP-2 will thus produce a destructive phenotype, causing fibronectin fragmentation, induction of apoptosis, or suppression of osteoblast differentiation. Several studies have demonstrated that *Porphyromonas gingivalis* may, in addition to gingival pain, activate the secretion of MMP-2 [36]. It also increases the migration of monocytes by activating the expression of MMP-9, which will indirectly lead to tissue destruction. Since, compared to MMP-2, MMP-9 has been shown to exert an increased activity and is largely present in periodontal patients, MMP-9 could be considered a predictor of disease activity [7]. Indeed, a decrease in the levels of MMP-1, -8, -9, -12, and -13 in the crevicular fluid has been observed following the treatment of aggressive periodontitis by descaling and surface enhancing, alongside antibiotic treatment. This demonstrates that the levels of MMPs are in a dynamic balance with the state of hygiene and health of periodontal tissues. Therefore, the presence of these proteases may facilitate the installation of a destructive microenvironment in the periodontium, which would determine the manifestation of the periodontal disease [17,30]. MMP-1, -8, and -13 have been detected in the peri-implant sulcular fluid, and this is associated with increased activity of annual vertical bone loss. In these cases, MMP-8 can be considered a possible marker for progressive bone loss in implants [37,38]. 

The levels of matrix metalloproteinases from the gingival crevicular fluid of patients with periodontal disease have also been studied from a diagnostic and prognostic point of view. For example, a cross-sectional study by Ramseier et al. found that salivary concentrations of MMP-8, -9, and orthopantomograms, combined with the presence of bacteria, can predict periodontal disease [39]. GCF levels of MMP-9 and -13 have been suggested as useful biomarkers for the progression of periodontitis in patients with moderate chronic periodontitis who have active sites and who have been observed for 2 months. These findings are in line with other cross-sectional studies showing that GCF levels of MMP-8 and -9 are correlated with disease activity in patients with chronic periodontitis [40,41]. Interesting data also emerged from a clinical study with 28 patients with chronic periodontitis and 22 controls [42,43]. The authors reported higher plasma levels of MMP-3, -8, -9 in patients with chronic periodontitis compared with controls, which decreased significantly 3 months after non-surgical periodontal treatment. Kinane et al. [44] also reported that the GCF levels of MMP-8 decreased significantly 3 months after non-surgical periodontal therapy in 20 patients with chronic periodontitis. Persistent increase of MMP-8 in GCF samples is considered a high risk of poor response to periodontal therapy [45]. Moreover, significant positive correlations were detected between MMPs-8 and -9 activities in GCF and periodontal disease severity, along with negative correlations with TIMP-1 and -2 levels. Therefore, a chairside MMP-8 test would be advisable to effectively differentiate clinically healthy sites and gingivitis from chronic periodontitis and also to effectively monitor the treatment of patients with chronic periodontitis [46]. 

When it comes to bone resorption, MMP-9 is probably the most important proteinase involved in this process because osteoclasts express this enzyme at an extremely high level. However, there are conflicting reports about the specific role of MMP-9 in bone resorption. 

For example, some studies have suggested that MMPs have, at best, a very small contribution to osteoclast bone resorption activity and that the selective MMP-9 inhibitor, TIMP-1, did not show a significant inhibitory effect on osteoclastic bone resorption [47], while other studies have shown that MMP-9 may play a key role in bone resorption caused by osteoclasts and that patients with MMP-9 genotypes, in association with their soluble protein, may have an increased risk of developing chronic periodontitis [36,45]. Application of orthodontic treatment can impact levels of MMPs. For example, orthodontic forces on a tooth can generate tension and compression in the periodontal ligament, which affect the remodeling of the alveolar bone and gingival tissue and are associated with high levels of MMP-1, -2 -8, and -9. Chemical inhibition of MMP-9 reduces orthodontic dental movements. Gingival hypertrophy without signs of inflammation can be caused by the reaction of the gingival tissue to the mechanical stress induced by orthodontic forces, triggering the activity of MMP-9, which makes it a good marker in the gingival fluid and gingival tissue in this situation. MMP-9 levels are higher in chronic gingivitis but lower than in the presence of active periodontal disease [48,49].

## 4. Matrix Metalloproteinase 8 (Collagenase 2)

### 4.1. General Aspects

The name collagenase denotes an enzyme capable of splitting tri-helical collagen. A collagenase isolated from human polymorphonuclear leukocytes (PMNLs) was first described in 1968 and was called PMNL-collagenase or neutrophil collagenase. Part of the extracellular matrix neutrophil collagenase is also called matrix metalloproteinase 8 (MMP-8) [50]. At first, it was thought that this collagenase is expressed only in neutrophilic leukocytes, but its enzyme and messenger RNA were also found in cells such as normal human joint chondrocytes, mononuclear fibroblasts, human endothelial cells, and human odontoblasts in bronchial epithelial cells [16], and it is the major collagenase in human dentin. It is expressed during early development in neuronal crest cells and adult melanoma cells but has also been found in cells in oral squamous cell carcinoma. Following the discovery of a third form of human collagenase (MMP-13), neutrophil collagenase became commonly referred to as collagenase 2 [26]. Neutrophil collagenase is stored intracellularly as a latent proenzyme in specific granules of polymorphonuclear leukocytes. Procollagenase activation occurs in the extracellular space after secretion and can be stimulated by stromelysin, trypsin-2, cathepsin G, or other mediators. The activated enzyme is able to split tri-helical collagen type I, II, and III and has several proteolytic properties, including hydrolysis of natural substrates such as gelatinous peptides, fibronectin, proteoglycans, fibrinogen, and aggrecan cartilage, as well as serpine inhibitors such as human C1 inhibitor or α1-proteinase, β-casein, and human chemokines. The enzymatic activity of MMP-8 is inhibited by TIMPS and α2-macroglobulin [51]. The gene for human neutrophil collagenase is located on the long arm of chromosome 11. In addition to their main phagocytosis function, human PMNLs have a high capacity for infiltration into the connective tissue. This is often associated with a defect in the extracellular matrix, especially during pathological processes such as inflammation from rheumatoid arthritis or osteoarthritis but also from periodontal disease, and this is initiated by collagenase in human neutrophils. The MMP-8 expression is stimulated by IL-1β and inhibited by insulin-like growth factor 1 [50,52]. 

The enzyme stored in specific granules is released from neutrophils as latent procollagenase under the action of various stimuli, such as interleukin 1 and 8, tumor necrosis factor α (TNF α), chemotactic formylpeptides, human anaphylatoxin C5a, fibrinogen- and fibrin-derived products, granulocyte-macrophage colony-stimulating factor (GMCSF), and calcium ionophore A23187. Several secreted forms have been described [53]. The mesenchymal cells of the dentin and pulp express and activate MMP-8, which is inhibited by TGF-beta 1 [54]. The precise pathway of the in vivo activation after secretion remains unclear, although various agents have been reported to initiate the in vitro activation according to the “cysteine switch” activation mechanism. The activation may also be accomplished through autocatalysis by compounds containing mercury, oxygen radicals, hydrogen peroxide or hypochlorite, or sodium thialate [55,56].

### 4.2. MMP-8 in Periodontal Diseases

Because type I collagen is the major component of the periodontal extracellular matrix, special attention has been paid to the role of collagenases, especially MMP-8 and MMP-13 and gelatinases, MMP-2 and MMP-9, in periodontal diseases. In fact, MMP-8 (or collagenase 2) has been referred to as one of the most promising biomarkers for periodontitis in oral fluids [32]. Several MMPs have been detected in various enzymatic forms in the gingival tissue, gingival crevicular fluid (GFC), and saliva, with the main metalloproteinases involved in the destruction of dental tissue being MMP-8, MMP-13, and MMP-9 [21,34]. MMP-8 and MMP-9 are the most abundant MMPs in periodontal tissues, and their level reflects the severity of the disease and its progression and response to treatment. They are secreted due to the infiltration of polymorphonuclear leukocytes and also macrophages, plasma, and residual cells such as fibroblasts, endothelial cells, keratinocytes, and bone cells [57].

Proteolytic cascades can lead to extensive destruction of the periodontal tissue due to the activation of MMPs and could be an interesting target for diagnosis and therapy. For example, MMP-13 is able to induce proMMP-9 activation and automatic activation of MMP-13 by in vitro auto-proteolysis. MMP-14 can activate MMP-8 and -13 in vitro, as well as MMP-2, and has been correlated in vivo with MMP-13 activation in periodontal sites [19]. Overall, these mechanisms could increase the destruction of periodontal tissues and could lead to the consequent progression of chronic periodontitis. In addition, nonproteolytic oxidative activation of MMP appears to be essential in periodontal inflammation. Reactive oxygen species (ROS) are cable of activating key MMPs in periodontal tissues through direct enzymatic oxidation but also through indirect mechanisms. Neutrophil degranulation is stimulated by periodontopathogenic bacteria, cytokines, and prostaglandins, leading to the release of myeloperoxidase (MPO) from the primary granules [58].

An important biological function of MMP-8 in the periodontium is to facilitate the migration of leukocytes, especially neutrophil granulocytes, from the circulation to the periodontal sulcus by the cleavage of collagen and other components of the extracellular matrix [59]. In addition, after the onset of inflammation, other cell types (e.g., fibroblasts) may also express MMP-8 [32]. Increased expression, release, and activation of uncontrolled MMP-8, along with other MMPs and proteinases, are thought to induce the inflammation associated with tissue destruction in periodontal disease and also other inflammatory diseases [34]. The activation of MMP-8 is facilitated by other MMPs and host proteases and increased oxidative stress caused mainly by neutrophil-released myeloperoxidase (MPO). Bacterial-derived proteases, such as Porphyromonas gingivalis (gingipain) and Treponema denticola, can also activate MMPs [39]. MMP-8 is currently considered one of the most promising biomarkers used for the anticipation, diagnosis, prognosis of treatment, and classification of periodontal disease [40]. Several studies have reported significant increases in MMP-8 levels in subjects with chronic periodontitis and periodontitis associated with diabetes [26]. For example, increased MMP-8 levels in GCF were reported due to the presence of periodontal pathogens such as *T. denticola* and T. forsythia, which represents a cascade of host responses induced by these organisms [7]. Effective periodontal treatment and MMP inhibitory adjuvant drugs have been shown to have an inhibitory effect in the progression of periodontal disease by reducing the level of MMP-8 in GCF and saliva [6]. MMP-8 activity was also found to be altered in various organs and body fluids in smokers [60] since tobacco induces degranulation in neutrophils and the growth of proinflammatory mediators that can influence the expression of MMP-8 in the periodontal environment of smokers. However, not all studies were successful in associating these levels of MMP-8 with smoking and the risk of periodontal disease [46,61]. Several available collagenase inhibitors have been approved by the US Food and Drug Administration (USDA) and are analogs of tetracycline and doxycycline hyclate [62]. As such, subantimicrobial doses of doxycycline (SDD) have been widely accepted as an important adjuvant therapy in the treatment of periodontitis, and several studies have already shown its effectiveness. 

## 5. Matrix Metalloproteinase 9 (Gelatinase B)

### 5.1. General Aspects

MMP-9 is a proteolytic enzyme that decomposes type IV collagen, which is the basic structural component of the basement membrane. MMP-9−1562 C/T SNP, located on chromosome 20q11.2-q13.1, is a metalloproteinase investigated for its association with an increased risk of developing cancer, emphysema, and other diseases. Based on the evidence from previous studies, the suggested mechanism behind this association could be that MMP-9 expression is primarily controlled at the transcriptional level, where the MMP-9 gene promoter responds to various stimuli such as cytokines and growth factors [49]. Moreover, the T allele of this variant can eliminate a binding site for a transcription repressor, altering the activity of the MMP-9 promoter, causing increased MMP-9 expression. In addition, changing the C-to-T site at position 1562 may alter nuclear protein binding in this region, resulting in increased macrophage transcriptional activity.

MMP-9 (or the gelatinase B) participates in the breakdown of various proteins from the connective tissue, including collagen type IV, V, and XI, proteoglycans, and elastin and is abundantly expressed in chronic periodontitis (CP) [63]. Various cell lines, such as polymorphonuclear leukocytes, macrophages, keratinocytes, fibroblasts, osteoclasts, eosinophils, and neutrophils, have been linked to the expression of the MMP-9 gene, located on chromosome 20q11.2-13.1. Genetic variations in the promoter region of the MMP-9 gene may have an effect on the transcription and synthesis of its proteins, which may influence the degradation of connective tissue of the protein and thus contribute to genetic susceptibility to periodontal disease [9]. Notwithstanding several studies examining the association of these polymorphisms with CP susceptibility and/or disease severity, the results thus far indicate a high degree of variability [62].

### 5.2. MMP-9 and Its Relationship with Periodontal Disease

Among the MMP isoforms, MMP-9 has been validated in various preclinical models as one of the most common mediators present in the stages of inflammation progression in patients with periodontitis. MMP-9 has been shown to be released by vascular macrophages upon exposure to pathogenic bacteria or during the host response and has been validated as a subclinical marker of vascular homeostasis [14,26]. MMP-9 has been found to regulate some mediators during the early stages of inflammation, including IL-1, -6, and -8, and prostaglandins [18,29]. Preliminary evidence has shown that MMP-9 expression is associated with damage to periodontal tissue during the active stages of periodontitis [37]. In this regard, some studies have shown that MMP-9 is increased in the gingival crevicular fluid during the initial phase of periodontitis, having a key role in the neoangiogenesis associated with the host response to periodontal pathogens [64]. The main source of MMP-9 is the polymorphonuclear neutrophils, with high levels being expressed in inflamed junctional and gingival epithelial cells in advanced periodontitis [20]. Thus, high MMP-9 levels may accurately reflect the condition of patients with periodontitis and can be a useful biomarker for diagnosis [65]. 

During periodontitis, it has been hypothesized that MMP-9, together with CRP (C-reactive protein), may inhibit the synthesis of nitric oxide (NO), which, in turn, may adversely affect the endothelium and the arterial vascular tone and eventually lead to endothelial dysfunction and an increased risk of cardiovascular disease [66]. Indeed, several studies showed a reciprocal relationship between endothelial damage due to NO release and serum levels of MMP-9. In addition, periodontal disease has been closely linked to high levels of NO, indicating a direct correlation between serum NO, MMP-9, and periodontitis. During the development of periodontal disease, the regulation of serum NO levels is related to the immune response of the host, which occurs as a result of infection with pathogenic periodontal bacteria [67]. Notwithstanding these findings, there is currently no full consensus regarding the effects of NO and direct oxidative stress on periodontal tissues. Indeed, some studies have shown high levels of NO and MMP in patients with periodontitis in the active phase of the disease, while other evidence has shown low levels of NO and some classes of MMPs during the development of periodontal disease [68]. A possible explanation for the results of this study comes from other evidence showing that MMP-9-mediated immune response is associated with the presence of heat shock proteins released during periodontitis, exerting a specific action on T lymphocytes [56]. In this regard, recent studies have validated MMP-9 as an essential modulator of host defense mechanisms during the initial immune phase, which, in turn, can trigger a cascade of events involving the entire host defense mechanism, the endothelial homeostasis, leading to an increased risk of developing periodontal disease [36,39]. Thus, targeted therapy focused on inhibiting MMP activity could be an ideal therapeutic option in the treatment of periodontal disease, in addition to scaling, root surfacing, and bone surgery. 

## 6. Matrix Metalloproteinase 13 (Collagenase 3)

### 6.1. General Aspects

In 1994, a new human matrix metalloproteinase with the structural features of a collagenase was identified and named collagenase 3 or metalloproteinase-13 (MMP-13). MMP-13 is a proteolytic enzyme belonging to a large family of endopeptidases responsible for extracellular matrix degradation, and it is characterized by the binding of zinc to their catalytic site [69]. The MMP-13 gene is located on chromosome 11q22.3, like other MMP genes. Thus, MMP-13 is a metalloproteinase secreted in the form of a proenzyme and includes (made up of) 471 amino acids. The activated form of MMP-13 has a catalytic domain and a domain similar to the hemopexin responsible for the degradation properties of MMP-13. Although the catalytic domain of MMP-13 itself can even degrade collagen, it is not as effective as the domain of hemopexin. MMP-13 is a very important metalloproteinase during skeletal growth and long bone maturation, as the MMP-13-mediated degradation of pre-existing extracellular matrix proteins has proved to be a necessary and important step in bone development prior to neoangiogenesis and mineralization. MMP-13 is oversynthesized in various pathological conditions, being involved in the degradation of collagen, aggrecan, fibronectin, and tenascin as well as other extracellular matrix proteins. Thus MMP-13 has an essential role in the progression of human carcinoma and metastatic processes, the development of acute articular rheumatism, and osteoarthritis [70]. As such, MMP-13 is overexpressed in the cartilaginous tissues of patients with osteoarthritis, and an increased level of MMP-13 in chondrocytes may be an initial mechanism in the development of osteoarthritis [19]. Although, initially, MMP-13 was thought to be revealed in the connective tissue, especially in developing cartilage and bone, the epithelial tissue and neuronal cells also contain MMP-13.

MMP-13 demonstrates versatility in the use of its substrate. In addition to being very active on type II collagen, MMP-13 breaks down other substrates, mainly extracellular matrix macromolecules, but also molecules such as the connective tissue growth factor (CTGF) and fibrinogen. MMP-13 is controlled at several levels: by controlling expression/synthesis but also by activating/inhibiting the active form of the enzyme. Unlike other metalloproteinases, the human MMP-13 gene can be transcribed into proteins with different activities and functions. A proteolytic cascade that includes MMP-14 and MMP-2 is activated by MMP-13. Different agents may regulate the MMP-13 transcription, especially growth factors, proinflammatory cytokines, and mechanical stimuli. Studies have shown that MMP-13 is a metalloproteinase much more complex than originally thought. Although our understanding of the biochemistry and regulation of MMP-13 has advanced greatly over the years, much remains unknown [71]. Although MMP-13 (collagenase 3) was first discovered in breast cancer [72], it remains a metalloproteinase with a major involvement in inflammatory diseases such as rheumatoid arthritis and osteoarthritis, where it is associated with the resorption and destruction of bones and cartilage [73]. This metalloproteinase is also expressed by various periodontal cells and inflammatory cells in association with chronic periodontal disease [74].

### 6.2. MMP-13 and Its Relationship with Periodontal Disease

The main cells where MMP 13 was detected were: fibroblasts, osteoblasts, macrophages, plasma cells, and gingival epithelial cells. MMP-13 has also been found to be involved in periodontal tissue destruction and alveolar bone resorption, along with MMP-9. The mechanisms of MMP regulation may be different, depending on the specific tissue and microenvironment. During the development of periodontal disease, these MMPs can be activated independently or together with pathogens and host proteases. [36]. Previous studies have shown increased concentrations of MMP-13 in saliva, especially in females, which have been associated with an increased attachment loss. The detection of MMP-13 in gingival fluid showed increased levels of this proteinase in patients with chronic periodontitis compared to healthy subjects. In saliva, however, the MMP-13 amount was high in localized periodontitis but low in generalized periodontitis [37].

MMP-13 has been associated with the destruction and resorption of bone and cartilage in periodontitis, being responsible for the activity of osteoclasts. Several mechanisms have been reported, including osteoclast-secreted proMMP-9 activation, which will further digest the denatured collagen derived from the MMP-13 activity; cleavage of galectin-3, a known inhibitor of the osteoclastogenesis expressed on the surface of osteoclasts, which results in the abrogation of its inhibitory effect; and, last but not least, by adjusting the RANKL/osteoprotegerin (OPG) axis, thus favoring RANKL and TGF-β1 signaling. The conclusion of previous research was that MMP-13 is involved in osteoclast differentiation but also in breast cancer and bone metastases. [75].

MMP promoter activities can be stimulated by various proinflammatory cytokines, growth factors, the extracellular matrix metalloproteinase inducer (EMMPRIN), and bacterial virulence factors. Periodontitis-specific cells, i.e., gingival fibroblasts, epithelial cells/keratinocytes, osteoblasts/osteoclasts, periodontal ligament cells, together with recruited inflammatory cells, i.e., the neutrophils, monocytes/macrophages, and plasma cells, can thus be stimulated or diminished by various cytokines. For example, transforming growth factor (TGF-β) can suppress the transcription of MMP-1, -3, and -8 genes, but it can also induce MMP-13 gene expression. Pathological growth and activation of MMPs can trigger a chain cascade of proinflammatory factors in the gingival tissue affected by periodontitis and in the gingival crevicular fluid. Furthermore, MMP-13 (collagenase-3) expression in gingival tissue sections was significantly increased in patients with chronic periodontitis, suggesting that MMP-13 expression is important in the proliferation of periodontal damage, in the progression of attachment loss, and in the deepening of periodontal pockets [76].

MMP-13 is capable of inducing proMMP-9 activation and MMP-13 self-activation by in vitro auto-proteolysis. In vitro studies have also shown that MMP 9 can be activated by proMMP-2 and proMMP-13 [77]. MMP-14, on the other hand, could activate MMP-8 and -13 in periodontitis sites. Thus, all these mechanisms could lead to an increase in the degradation of periodontal tissue and, consequently, to the progression of chronic periodontitis. In addition, nonproteolytic oxidative activation of MMPs appears to play a central role in periodontal inflammation. At the level of periodontal tissues, reactive oxygen species (ROS) are able to activate key MMPs by oxidation of the enzymes using both direct and indirect mechanisms. Periodontopathogenic bacteria, cytokines, and prostaglandins lead to the degranulation of neutrophils, resulting in the release of myeloperoxidase (MPO). In addition to its antimicrobial activity, MPO is involved in regulating catabolism and connective tissue degradation by altering the protease/anti-protease balance. Previous research has shown another possible mechanism involved in the development and progression of periodontal disease, namely, the activation of MMP mediated by reactive oxygen species (ROS). Oxidative stress increases the turnover of the extracellular matrix mediated by MMP-2, -9, and -13 in fibroblast and tumor cells [56]. Although there is considerable research on the importance of MMP-13 in the progression of periodontal disease, the exact mechanisms of association of periodontitis with this metalloproteinase are still unclear. 

## 7. MMPs Inhibitors and Biomarkers in Periodontal Disease

Among other molecules, the MMP inhibitors adopted in periodontal therapy are modified tetracyclines. Tetracyclines are antibiotics capable of inhibiting connective tissue degradation. Inhibitors are obtained by the chemical modification of molecules in the tetracycline family after the separation of antibiotic and protease inhibitor activities [78]. Indeed, serum levels of MMPs can be lowered with the administration of sub-antimicrobial doses of antibiotics such as doxycycline or tetracycline. For example, tetracycline (CT) can reduce MMP-8 and -9 activity in GCF and gingival tissue, even at a much lower dose than a traditional antimicrobial dose used in conventional therapy [35,39]. Minocycline provided the first evidence of inhibiting MMP by CT and its derivatives in the oral environment since it inhibits the collagenolytic activity of gingival crevicular fluid in the absence of bacteria. The CTs have cationic chelating properties that inhibit MMP activity in the extracellular environment and prevent the activation of proMMP through oxidation by eliminating the reactive oxygen species [79]. The TC also decreases transcriptional levels of MMP at an intracellular level.

Doxycycline hyclate is a low-dose tetracycline analog lacking antimicrobial activity, and it is indicated for the treatment of periodontal disease, acting by inhibiting the mechanisms of the MMP-8 and MMP-13 protease. The therapeutic effect of this antibiotic is due to the modulation of the host response since low-dose formulas do not exert antimicrobial activity [80]. Tetracyclines have been found to inhibit MMP activity through cationic binding proteins, and their use in combination with mechanical periodontal therapy is widely accepted. In adults with periodontitis, low-dose doxycycline is currently used as adjunctive therapy in order to inhibit MMP activity because it significantly reduces the severity of periodontal disease, including alveolar bone loss. Chemically modified tetracyclines (CMTs) do not have the 4-dimethylamino group of TC, which exerts antibacterial activity. Several CMTs with a range of potency and specificity for MMP’s inhibition have been developed [81]. For example, CMT-3 and -8 are the most potent inhibitors of MMP, with collagenase and CMT-3 being the only CMT with demonstrated efficiency against MMP-1 [82]. It has also been found that various MMPs are inhibited by chlorhexidine (CHX). CHX is a biguanide chemical substance that provides effective antiseptic effects and is used to control plaque and reduce gingival inflammation. Several studies have shown that chlorhexidine directly inhibits MMP-2, MMP-8, and MMP-9, probably through a chelating mechanism. CHX has been shown to dose-inhibit the collagenolytic activity of MMP-8 released by the human polymorphonuclear leukocytes triggered by forbol-12-myristate-13-acetate (PMA) [83]. Taken together, these findings show that analysis of salivary levels of various risk mediators, such as metalloproteinases, are promising approaches used for the detection of early stages of various pathologies, both oral and systemic.

**Figure 3:**
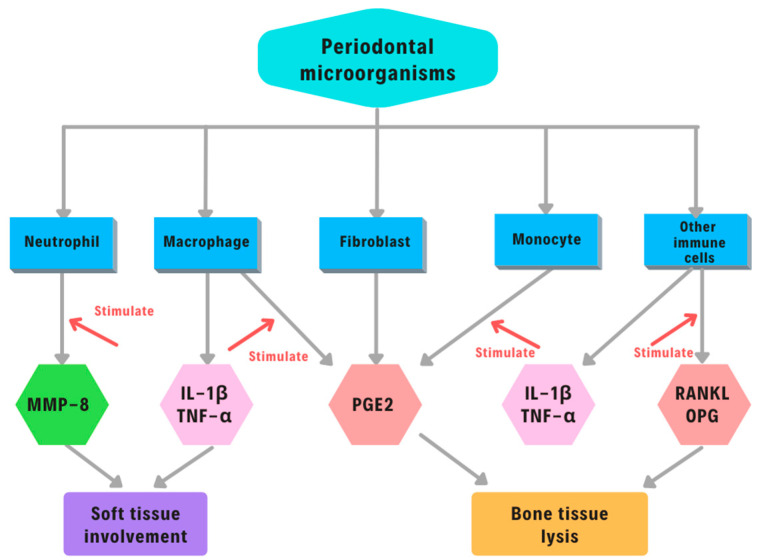
Biomarkers in periodontal disease. Abbreviations: IL-1 β, interleukin -1; TNF-α, tumor necrosis factor α; PGE2, prostaglandin E_2_; RANKL, receptor activator of nuclear factor kappa-Β ligand; OPG, osteoprotegerin.

There are numerous biomarkers involved in the complex pathological mechanism of periodontal disease (Figure 3). 

For example, TNF-α is part of the major inflammatory cytokines, which are typically produced at the inflammation site by infiltration of mononuclear cells. TNF-α is a pleiotropic cytokine that can improve the host’s defense mechanism by mediating inflammation and increasing cellular immune function [84,85]. At the same time, it can induce various pathological conditions due to TNF-α toxicity, causing tissue damage (septic shock syndrome, cachexia, autoimmune diseases, rheumatoid arthritis. meningococcal sepsis) [84,85]. Salivary TNF-α levels can represent a valuable and accurate marker, both for aggressive and chronic periodontitis. Salivary PGE2 and IL1β can be considered essential mediators that play a key role in the pathogenic process of periodontal disease. They are strong stimulators of bone resorption and are produced by the cells of the human periodontal ligament in response to mechanical stress. PGE2 not only mediates inflammatory responses such as the increase of vascular permeability and dilation but can also work as strong stimulators of bone resorption and formation. This dynamic mechanism can be influenced by the concentration of PGE2 [86]. On the other hand, the RANK/RANKL/OPG system plays a significant role in the activation of osteoclasts, which are well known for their capacity to upregulate bone resorption [87]. However, periodontitis cannot be distinguished between mild, moderate, and severe forms based exclusively on RANK/RANKL/OPG, but it may be used as a complementary diagnostic tool for early diagnosis [87].

## 8. MMPs and Peri-Implantitis

Dental implants are increasingly used for partial or complete edentulism. Peri-implantitis and peri-mucositis, two pathological conditions that affect tissues surrounding dental implants, are highly prevalent, representing approximately 25% and 43%, respectively, of patients with implants [88]. They are characterized by inflammation of the peri-implant connective tissue, leading to the progressive loss of supporting alveolar bone, which can cause more destructive lesions than periodontitis [85]. As such, peri-implant sites show large inflammatory processes with increased M1 type tissue destructive macrophages and neutrophils and high levels of leukocyte-type collagenases such as MMP-8 in the peri-implant sulcular fluid (PISF). This led to the development of point-of-care tests using MMP-8 as a reference test for grading peri-implantitis. Low MMP-8 levels were associated with periodontal and peri-implantitis health while the upregulation of MMP-8 levels denoted an increased risk for inflammation [89]. The MMP-8 tests proved to be more sensitive and reliable than other biomarkers such as calprotectin, gelatinases, neutrophil elastase, myeloperoxidases and MMP-9, although PISF MMP-9 also is a good marker for tissue health surrounding the implant [90]. Both MMP-8 and MMP-9 levels were associated with the degree of healing process and osseointegration, indicating their functional role in tissues around the implant. For example, higher levels were detected during or immediately after implantation compared to post-implantation and healing [91]. Although MMP-8 is considered as the main collagenase in active peri-implantitis [92], MMP-9 has also been shown to be involved in the pathogenesis of peri-implantitis [93] via a LOX-1 (lectin-like oxidized low-density lipoprotein) and Erk1/2 (extracellular signal-regulated protein kinase) mechanism that has been suggested as a potential target for decreased inflammation and increase apoptosis in peri-implantitis. In addition, MMP-1 may be involved in the pathogenesis of peri-implantitis, given its increase in fibroblasts from peri-implantitis [94] and reduced gene expression of tissue inhibitors of matrix metalloproteinases (TIMP-1) [32], which may indicate a loss of attachment around dental implants [95]. It is interesting that a polymorphism in the MMP-1 promoter (G-1607GG) involved in transcription has been reported in implant failure [96]. A similar polymorphism for the promoter region of the MMP-8 gene (C-7997) [97] and MMP-13 (-77 A < G) has been associated with loss of osseointegration and implant loss [98].

## 9. Conclusions and Perspectives

Periodontitis is an inflammatory disease that is modulated by several factors, among which members of the MMP family play an important role in the degradation of the extracellular matrix and the destruction of periodontal tissue. Due to their particular physiopathological pattern, MMPs are strong predictors for periodontitis. Although different opinions exist regarding the accuracy of salivary MMPs levels versus those determined from the crevicular fluid in relation to periodontal pathology, recent evidence shows that both methods can be extremely useful for a precise diagnosis and efficient follow-up treatment. Nonetheless, recent evidence suggests that salivary MMP-9 seems to be a more sensitive marker for periodontal inflammation during orthodontic treatment, which opens new perspectives and approaches in reducing periodontal hazards during such treatments. At the same time, chairside determination of crevicular MMP-8 might represent a rapid and accurate diagnostic method to significantly reduce the failure rate of dental implants. Both MMP-8 and MMP-9 are valuable diagnostic tools in treating periodontitis or peri-implantitis, while MMP-13 affects the activity of osteoclasts and bone resorption, thus contributing to the destruction of the periodontal tissue. It is also interesting that MMPs gene polymorphism has been linked with the risk of periodontitis. For example, MMP-9-753 C/T polymorphism lowered the risk of chronic periodontitis, while MMP-3-1171 5A/6A and MMP-8-799 C/T polymorphisms increased the risk of chronic periodontitis [99]. Thus, a better understanding of the underlying pathophysiological mechanisms of metalloproteinases in association with the presence of periodontal or peri-implant damage could lead to novel diagnostic and therapeutic methods of prevention, management, and treatment.

## Figures and Tables

**Table 1:** The main metalloproteinases: name, substrates, and the main diseases in which they are involved.

Type of MMP	Name	Substrate	Production	Physiological Function	Associated Disease’s	References
MMP-1	Collagenase1/Interstitial Collagenase/Fibroblast Collagenase	Collagen I, II, III, VII, VIII, X, XI, Gelatin, Fibronectin, Aggrecan, Entactin, Tenascin, Ovostatin, Casein	Fibroblast, Keratinocytes, Endothelial cells, Macrophages, Osteoblast, Chondrocytes, Platelet	Wound healing, re-epithelialization, cell proliferation, Keratinocyte migration	Periodontitis Rheumatoid arthritis, Atherosclerosis, Fibrosis, Autoimmune disease, Cancer	[14,17,18]
MMP-2	Gelatinase A/72-kDa type IV collagenase	Collagen, Elastin, Endothelin, Fibroblast growth factor, MMP-9, MMP-13, Plasminogen, and TGF-β,	Cardiomyocytes, Fibroblasts, and Myofibroblasts.	Neovascularization, Angiogenesis, Promoting and inhibiting Inflammation,	Cancer, asthma, lung diseases,	[19,20]
MMP-8	Collagenase2/Neutrophil Collagenase	Collagen I, II, III, Fibronectin, Aggrecan, Ovostatin	Chondrocytes, Endothelial cell, Macrophages, Smooth muscle cell	Periodontal tissue turnover, Anti-inflammatory activity, Wound healing	Periodontitis, Rheumatoid arthritis, Asthma, Cancer	[19,21,22]
MMP-9	Gelatinase B/ 92-kDa type IV collagenase	Gelatin, Type V collagen, Laminin, Fibronectin	Neutrophils, Eosinophils, Epithelial cells	Wound healing, Embryo implantation, Neovascularization, immune cells function, tissue remodeling	Arthritis, Metastasis, Pulmonary disease, Infections, Cardiovascular disease, Periodontal disease	[20,21]
MMP-12	Macrophage elastase	Elastin, Laminin, Fibronectin, Vitronectin, Type IV collagen	Endothelial cells, Neutrophils, Fibroblasts, T-cells, Myocytes, Macrophages,	degrade extracellular matrix component	Emphysema, Arthritis, Cancer, Periodontal disease	[8,23]
MMP-13	Collagenase 3	Collagen I, II, III, IV, IX, X, XIV, Fibronectin, Laminin, Gelatin, Aggrecan, Plasminogen, Osteonectin	Epithelial cell, Neuronal cell, Connective tissue (Cartilage and Bone)	Osteoclastic activation, Anti-inflammatory activity	Periodontitis, Osteoarthritis, Liver fibrosis, Cancer	[19,24,25]

## Data Availability

Not applicable.
